# Device Modeling of Organic Photovoltaic Cells with Traditional and Inverted Cells Using s-SWCNT:C_60_ as Active Layer

**DOI:** 10.3390/nano12162844

**Published:** 2022-08-18

**Authors:** Vijai M. Moorthy, Viranjay M. Srivastava

**Affiliations:** Department of Electronic Engineering, Howard College, University of KwaZulu-Natal, Durban 4041, South Africa

**Keywords:** carbon nanotube (CNT), bulk hetero-junction structure, device modeling, organic solar cells, inverted organic solar cells, microelectronics, nanotechnology, VLSI

## Abstract

This research work presents a thorough analysis of Traditional Organic Solar Cell (TOSC) and novel designed Inverted OSC (IOSC) using Bulk Hetero-Junction (BHJ) structure. Herein, 2D photovoltaic device models were used to observe the results of the semiconducting Single Wall Carbon Nanotube (s-SWCNT):C_60_-based organic photovoltaic. This work has improved the BHJ photodiodes by varying the active layer thickness. The analysis has been performed at various active layer thicknesses from 50 to 300 nm using the active material s-SWCNT:C_60_. An analysis with various parameters to determine the most effective parameters for organic photovoltaic performance has been conducted. As a result, it has been established that IOSC has the maximum efficiency of 10.4%, which is higher than the efficiency of TOSC (9.5%). In addition, the active layer with the highest efficacy has been recorded using this material for both TOSC and IOSC Nano Photodiodes (NPDs). Furthermore, the diode structure and geometrical parameters have been optimized and compared to maximize the performance of photodiodes.

## 1. Introduction

Solar energy has achieved various advancements due to renewable energy sources in the last few decades. These devices provide long-term power at a low operating cost and low pollution. The photovoltaic effect is the underlying principle of this technology. When exposed to sunlight, solar cells convert that energy into electrical current. Based on the material configuration, organic cells are classified as third-generation solar cells [1]. Since their discovery over a decade ago, organic photovoltaic devices have attracted considerable attention as non-conservative renewable energy sources [2,3]. In 1960, Williams [4] had performed an experimental analysis on photovoltaic effects that occur at semiconductor electrolyte interfaces. The cadmium sulfide and various other compounds were studied using single-crystal specimens. The analysis exposes the photovoltaic effects of electrode materials when they chemically react with one another. Loferski [5] explained that the advances in quantum mechanics and semiconductor technology enabled a deeper understanding of photovoltaic effects and resulted in the creation of more advanced photovoltaic devices.

Goetzberger et al. [6] have reported that selenium solar cells have less than 1% conversion efficiency. Therefore, experiments were carried out on a variety of other materials, in which the majority met these criteria; however, the most effective photovoltaic devices used p–n junctions on silicon semiconductor materials [7]. The first solid-state solar cell was used in 1883 by coating a thin gold film over a selenium semiconductor to form a junction. Ohl [8] have analyzed a crystalline silicon sample with a crack in the middle that formed an electric current when exposed to light. This work paved the way for silicon PV technology, which is a Si p–n homojunction cell with top and bottom electrodes. A typical Si solar cell starts with a p-type substrate that was doped to form an n-type emitter on top. Then, phosphorus is diffused into the base substrate during n-type doping and screen printing creates the top and bottom electrodes. Printing solar cells is the simplest and most established way of producing them. Moreover, to enhance the device’s performance, the top surface of the cell has been coated with an anti-reflection coating.

Alferov [9] has reported that solar cells with p–n junctions are composed of Aluminum Gallium Arsenide and Gallium Arsenide (GaAs) hetero-junctions. The GaAs solar cells had only one flaw: They were expensive to produce. The Czockralski and the Bridgman methods were initially used to produce single crystals of GaAs. Due to the low efficiencies and imperfect crystals, these methods for material growth were not successful. Their threshold current densities have been achieved at (4~5) × 10^3^ A/cm^2^ at 300 K. Organic semiconductor-based OSCs are solid-state thin-film solar cells. Organic semiconductors are aromatic hydrocarbons with sp_2_ hybridized carbon atoms that have alternate single and double bonds, such as a semiconductor. These materials are known as conjugated semiconductors due to the change in single and double bonds between carbon atoms. According to Chen et al. [10], the conjugated polymers based on organic semiconductors are a substitute for inorganic devices due to their various favorable characteristics, including portability, adaptability, and solution process deposition at ambient temperature. The highest external quantum efficiency observed for the OSC BHJ solar cell was 0.836 for 500 nm wavelength and 0.815 for 450 nm IOSC BHJ solar cell.

Furthermore, Malti et al. [11] have claimed that solar energy efficiency improved through a number of initiatives. The device-level efficiency of white light has been improved to 5%. A maximum efficiency of around 8% is achieved when the band-gap of the cell is around 1.5 eV, *V_oc_* = 0.61, for 400 nm thickness at *T* = 300 K. Brabec et al. [12] have reported results contrary to inorganic technologies, which have higher efficiency. The ability to produce Organic Photo Voltaic (OPV) on low-cost substrates in reel-to-reel fashion with regular coating and printing methods is the primary characteristic that makes OPV significantly attractive. The OPV was discussed in terms of economic and technological aspects of manufacture. Yu et al. [13] have fabricated the OSC using C_60_ and its functionalized derivatives were used to mix with the semiconducting polymer. Herein, two orders of magnitude better than those that have been achieved by pure MEH-PPV devices were observed, due to the remarkable efficiencies of the materials used. As a result, significant efforts have been exerted to enhance the efficiency of OSCs by employing cutting-edge materials and designs, etc. This aim was achieved by *h*_c_ = 90% and *h*_e_ = 5.5% at 10 mW/cm^2^ for internal carrier collection efficiency and energy conversion efficiency.

Scharber and Sariciftci [14] have examined the BHJ solar cells and their basic principles of operation and device design. The commercial viability of BHJ solar cells depends on their ability to convert solar energy into electrical energy with high efficiency. The Power Conversion Efficiency (PCE) in the range of 10–15% was predicted by several models based on the best available materials and device architectures. Higher efficiencies were achieved if the Shockley–Queisser limit was considered. Inorganic photovoltaic systems that depend only on charge carrier recombination by radiation may be as efficient as BHJ devices that do not show this phenomenon. Marinova et al. [15] have presented an overview of the key processes in photovoltaic systems based on OSCs and Perovskite Solar Cells (PSCs), focusing on interfacial processes of engineering materials. Recently, the authors have employed poly (3-hexylthiophene-2, 5-diyl):phenyl-C61-butyric acid methyl ester (P3HT:PCBM) blends in polymer solar cells. A transparent conductive electrode material used in BHJ is Indium Tin Oxide (ITO). BHJ may be classified into two types: Traditional and inverted.

At a later time, Kumavat et al. [16] reported the development of solar cells, including some fundamental concepts used in Dye-Sensitized Solar Cells (DSSCs) and OSCs. The OSCs and DSSCs are two of the technologies mentioned in the review. Liang et al. [17] have examined the impact of Zinc Oxide (ZnO) surface modification, Common Buffer Layer (CBL) shape, composition/hybrids, thickness, nanostructures, and doping on the efficacy of inverted PSCs. This research assisted in the acquisition of further knowledge on the subject. The authors reviewed the most extensively used CBL, which is produced from pristine ZnO for improving PCE and long-term device stability. For high PCE in inverted PSCs, using a high-quality CBL material that allows for efficient electron collection and transport is critical. This is due to its excellent electron mobility, optical transparency, low-cost solution methods at low temperatures, a wide range of morphologies, and its environmental stability. ZnO material has been widely examined for CBL of inverted PSC. As established in this work, electronic processes occurring at the interface between the ZnO CBL and the active polymer layer considerably impact solar cell performance.

According to a subsequent publication, Zhao et al. [18] have stated that the IOSC based on active layer P3HT:PCBM material was composed of a Molybdenum Trioxide (MoO_3_) Hole-Transporting Layer (HTL) and very thin Calcium (Ca) Electron-Transporting Layer (ETL). Additionally, the cell was capable of working at high temperatures. Adding 1 nm of Ca to an ITO electrode changes the ITO work function, rendering it better suited to the extraction of electrons, with an achievement of 3.55% PCE. Zhao et al. [19] have designed an IOSC with an extremely thin Ca layer for electron transport, a photon-absorbing combination of P3HT and PCBM, and a performance-optimized MoO3 layer enabling hole transport. A MoO3 layer was inserted between the anode and active layer. According to the findings of the research, this increased the performance of the device significantly, with an achievement of 3.86% PCE for the optimized device. Waldauf et al. [20] have produced an effective electron selective bottom contact by covering ITO with solution-processed Titanium Oxide (TiOx), and then annealing the resulting structure. As an efficient carrier collecting network with low vertical phase segregation, o-xylene is employed in both conventional and IOSC applications. With the application of o-xylene, a carrier network was created, which is highly hygroscopic and transparent to light. These inverted layer sequence OPVs have PCE of more than 3%.

However, White et al. [21] have created an OSC, which is composed of ITO electrode with a P3HT/fullerene combination and a solution-processed ZnO interlayer between ITO and the active layer, with Silver (Ag) functioning as a hole-collecting back contact. When electron extraction by ZnO and hole extraction via Ag are both successful, with little open-circuit potential loss, a PCE of 2.58% was observed, along with minimum loss in open-circuit potential. Gilot et al. [22] have compared theoretical calculations using optical modeling to real findings for systems using ZnO as an optical spacer. The P3HT and PCBM mix showed good agreement. Another issue with organic materials is their weak absorption, which is primarily restricted to the visible range. Behjat et al. [23] have discovered that utilizing a ZnO layer as an optical spacer between the active layer and reflecting electrode allows the optical electric field (present within BHJ solar cells) to be re-distributed within the solar cell structure. ZnO film can be transmitted into the absorbing layer (the maximum level of the electric field) according to the theoretical analysis carried out using optical modeling. An optical spacer was used in this work to compare theoretical calculations to practical results. A substantial improvement in short-circuit current density was observed when a ZnO optical spacer layer was utilized. The PCE of the BHJ solar cell was increased by up to 3.49% due to the device’s electrical and optical properties.

Lee et al. [24] have demonstrated that TiOx may be used as an optical spacer in the BHJ structure. The findings showed that the TiOx optical spacer’s improved efficiency is inversely related to the amount of processing additive used. Surface roughness is produced on the BHJ film as a result of the manufacturing process, which reduces the efficiency of the optical spacer. Alternatively, active layer nanomaterials, such as CNTs and fullerene derivatives can be used to produce an efficient NPD.

Yang et al. [25] recently demonstrated that N,N-di(2-ethylhexyl)-6,6′-dibromoisoindigo and an amino-containing fluorene subunit were effectively combined to form the alcohol-soluble polymer PFN-ID for OSC using PTB7-Th:PC71BM as the active layer. The optimal PCE of 9.24% is achieved, which is 1.62 times greater than the devices without cathode interfacial layers. Xue et al. [26] employed the Methyl Salicylate (MeSA) in their study as a non-halogen additive for inverted OSCs, and its effects on the performance of the blend film and photovoltaic system were analyzed. The findings show that MeSA exhibits an efficiency of 9.45% and improved FF (>70%) with a 7% MeSA additive. Ren et al. [27] employed Tetrasodium Iminodisuccina (IDS), which is a non-conjugated small molecule added to the conventional devices based on PTB7-Th:PC71BM to serve as an Electron Transport Layer (ETL). The PCE of 9.45% is achieved with *V*_oc_ of 0.80 and FF of 70.01%.

In the previous research work, Moorthy et al. [28,29,30] have analyzed the BHJ and Planar Hetero-Junction (PHJ) topologies utilizing various nanomaterials and fabricating and testing the NPD device for the subretinal implant. The nanomaterials used improved the BHJ performance for the subretinal implant application.

To extend the author’s previous work [28,29,30], this present research work realizes the novel model of TOSC and IOSC BHJ. The authors analyzed the performance of both the TOSC and IOSC NPD cells using organic materials and device architecture on cell performance for various configurations with various light-absorbing materials and thin sheets to optimize the cell’s hole and electron transport layers. Molecular structures of s-SWCNT and C_60_ perform better than the other active layer’s blends, as reported in the authors’ previous work. An organic photovoltaic cell based on a single diode cell has been used in this work. 

This research work has been organized as follows: Section 2 presents the structure of the proposed photovoltaic devices, as well as the selection of active layer, ETL, HTL, and electrode materials. Section 3 discusses the methodology of PV cell structures, which is comprised of organic semiconductors, such as conjugated polymers (CP), as active materials and transparent electrodes. Section 4 provides a comparison, optimization, and evaluation of TOSC and IOSC NPD structures. Finally, Section 5 concludes our work and provides recommendations for future work.

## 2. Structures of TOSC and IOSC Organic Nano Photodiodes and Choice of Materials

**Figure 1:**
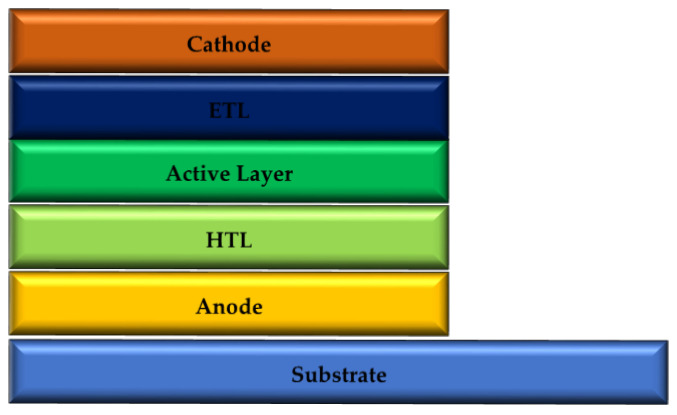
Structure of the TOSC.

To conduct this research, a passive OPV device has been used to transform light intensity into an electric current. Figure 1 shows the OSC fundamental structure. One of the primary goals of this research is to examine and compare how the TOSC and IOSC device architectures affect the performance of the device, depending on material properties and layer thickness. The second goal is to optimize the design to achieve maximum performance.

### TOSC and IOSC NPD Structure Using BHJ

A polymer, CNT, ITO, PEDOT:PSS, and aluminum materials are all used to design the TOSC and IOSC NPD structures. These NPD structures generate power under incident light. This section presents the structures of the proposed photovoltaic devices to be examined in this work and the selection of active layer, HTL, ETL, and electrode materials. The reasons underlying this choice of materials are as follows:

A. In this model, the ITO material has been used as a transparent electrode due to its various features. In the author’s previous works [28,29,30], graphene has been utilized as an electrode due to its biocompatibility and unique properties that suit the subretinal implant application. However, there were some shortcomings while fabricating the graphene-based OSC. Therefore, in this present analysis, the authors employ the ITO due to its electrical conductivity, optical transparency, simplicity of deposition as a thin layer, and chemical resistance to moisture. The film’s conductivity improves with thickness and charge carrier concentration, while its transparency diminishes. For applications demanding low-cost, large-area manufacturing, ITO is an attractive solution [31,32].
B. The PEDOT:PSS layer is commonly employed as an HTL in both OSC and DSSC. This is due to the fact that it smoothens the ITO electrode surface, which is critical in OSC and DSSC. Additionally, due to its strong electrical conductivity and excellent oxidation resistance, it is well suited for electromagnetic shielding and noise suppression applications. In the visible, near-infrared, and ultraviolet light spectrums, PEDOT:PSS-based polymeric films exhibit excellent transparency, with almost 100% absorption from 900 to 2000 nm. Moreover, they are transparent in the near-infrared and near-ultraviolet regions. However, PEDOT offers the conduction properties, and PSS (in contact with water) results in the formation of a hydrated colloidal solution [33].
C. The s-SWCNTs provide a unique combination of the solution processability, electrical tunability, and robust absorption of organic semiconductors with high charge mobilities, as well as outstanding chemical stability. Since they are strong optical absorbers with adjustable band-gaps, transfer energy and charge on ultrafast timescales are reasonably chemically stable. Additionally, solution-processable s-SWCNTs are attractive photo absorbers for next-generation photovoltaic solar cells and photodetectors. Due to these characteristics, s-SWCNT is the ideal donor material for this research [34,35].
D. Fullerene (60) or C_60_ is composed of 60 carbons, with 20 hexagonal and 12 pentagonal sections. Fullerene does not have a saturated state. Three carbons are linked together rather than four by the fused rings of carbons 5 and 6, so-called carbon 60 fullerenes. Fullerene is a molecule that can accept electrons. Depending on their properties, it can be employed in thermal evaporation systems as electron acceptors. Moreover, it can be used as an interface layer. C_60_ fullerene can be better as an electron acceptor since it is more compatible and can deliver suitable composites with the donor material [36].
E. One of the most common ETLs in PSCs and OSCs is Titanium Dioxide (TiO_2_). It is one of the widely utilized ETLs since it is very good at transmitting light in the visible range [37].
F. The work function of the cathode material should be lower than the anode material. The work function of aluminum is 4.2 eV, which is smaller than the ITO work function of 5.1 eV, and thus meets the criteria.

**Figure 2:**
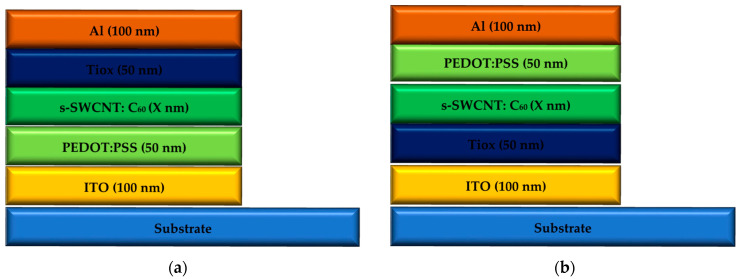
(**a**) Proposed TOSC and (**b**) IOSC structure.

The BHJ sandwiched between the ETL and HTL is a blend of donor and acceptor with discrete band gaps. The ITO and aluminum electrodes are two layers that play a role in the process of exciton generation, diffusion, and free-carrier generation processes. Detailed representations of the TOSC and IOSC BHJ structures considered in this work are shown in Figure 2a,b, respectively, along with the materials and layer thicknesses used in each structure.

Figure 2a depicts the TOSC NPD based on the BHJ structural configuration. Aluminum metal is used as the cathode, TiOx is used as the ETL, the electron donor is s-SWCNT, while the electron acceptor is C_60_ fullerene, since an HTL PSS is utilized and ITO is used as the anode. The BHJ-based TOSC is defined by sandwiching a blend of donor and acceptor between the ETL and HTL to form a junction. This limits the photoactive layer thickness and consequently the absorption effectiveness. The BHJ concept overcomes the PHJ device’s exciton diffusion barrier. Donor and acceptor materials are completely mixed in polymer BHJ. To achieve 100% efficiency in exciton separation, the mixed layer has created a donor-acceptor interface that is spatially distributed.

The TOSC BHJ solar cell is composed of the same materials as the IOSC NPD, with the exception that the ETL and HTL layers have been switched. Figure 2b depicts the IOSC BHJ NPD structure that has been realized in this research. Moreover, the TOSC structure is composed of the cathode aluminum metal, TiOx as ETL, electron donor s-SWCNT, electron acceptor C_60_ fullerene, PEDOT:PSS as HTL, and ITO as the anode. These materials are produced on a flexible/glass substrate, which improves the efficiency and transparency of the device by increasing its transparency. The structure is used for TOSC substrate/ITO/PEDOT:PSS (HTL)/s-SWCNT:C_60_ (blended active layer)/TiOx (ETL)/Al. For IOSC, the structure is inversed by swapping the HTL PEDOT:PSS and ETL TiOx. The IOSC is organized as follows: Substrate/ITO/TiOx (ETL)/s-SWCNT:C_60_ (blended active layer)/PEDOT:PSS (HTL)/Al. Each OSC and IOSC cell has two lighted sides, which are both placed on one side of the transparent ITO substrate. The existence of ETL and HTL collects electrons and holes at their respective electrodes and transports them to their corresponding destinations. The thickness of the different active layers has been explored in detail and contrasted between the two structures.

The purpose of this research is to design, analyze, compare, and contrast the TOSC and IOSC NPDs using BHJ structures. Various physical characteristics, such as the thicknesses of different layers and other factors, have been used to develop this structure. To determine the I–V characteristics of these NPDs, an electronic device simulator has been used. In this instance, the anode contact is supplied with a bias voltage; the voltage range is 0.01 to 0.09 V, with a 0.05 V step increase.

## 3. Device Design Methodology

To represent the charge carrier transit in the device, the model is used to determine all electrons, carrier continuity, and drift-diffusion hole equations in location space, which is a location space representation of the device. In addition, the model determines Poisson’s equation to estimate the electrostatic potential. The SRH formalism, established by Shockley–Read–Hall has been utilized to describe carrier trapping and recombination in the model [38]. Model parameters may be used to control the distribution of trap states, which allows for a more realistic representation of recombination and carrier trapping [39,40,41]. Organic semiconductors’ opto-electronic transport method relies on the Density Of States (DOS) due to their amorphous nature [42,43,44]. For transport and carrier trapping, this tool makes use of the finite-difference drift-diffusion model, which is based on finite differences.

To estimate the free-carrier transport, the BHJ material is treated as a single layer, with the band-gap energy defined by the *p*-type HOMO (highest occupied molecular orbital) and *n*-type LUMO (lowest unoccupied molecular orbital) energies [21]. In the organic materials, the HOMO level corresponds to the *E_v_* energy band-gap edge, whereas the LUMO corresponds to the *E_c_* energy band gap edge. Since the BHJ is a combination of *n*-type and *p*-type zones, the most straightforward approach is to represent it as an analogous layer with an equivalent band-gap energy between the *p*-type HOMO and *n*-type LUMO levels. The following are the basic equations employed in this model [38,39,40,41,42]:

Gauss’s Law:

(1) $$\nabla \in_{0}\in_{r}r\nabla \emptyset =q\left(n-p\right)$$

where *q* is the elementary charge on an electron, $\in_{0}$ and $\in_{r}$ denote the permittivity of free space and the relative permittivity of the active layer, respectively, *n* and *p* are the free electron and hole densities, respectively. For electrons and holes, the bi-polar drift-diffusion equations are solved in position space. These equations are solved to obtain the electron current flux density *J_n_* and the hole current flux density *J_p_*:

(2) $$J_{n}=q\mu_{e}n\nabla E_{c}+qD_{n}\nabla_{n}$$

(3) $$J_{p}=q\mu_{h}p\nabla E_{v}-qD_{p}\nabla_{p}$$

where *J_n_* and *J_p_* represent the electron current flux density *J_n_* and the hole current flux density *J_p_.* Here, the Maxwell–Boltzmann statistics for the free carriers are assumed, where *D_n_; p* denote the electron and hole diffusion coefficients.

To achieve this, it is necessary to conserve charge carriers by solving the charge carrier continuity equations for electrons and holes, which are illustrated in Equations (2)–(5), respectively. Electron continuity equation:

(4) $$\nabla .J_{n}=q\left(R-G+\frac{\partial_{n}}{\partial_{t}}\right)$$

Hole continuity Equation:

(5) $$\nabla J_{p}=-q\left(R-G+\frac{\partial_{p}}{\partial_{t}}\right)$$

where *R* and *G* represent the net recombination and generation rates per unit volume, respectively.

The above equations are used to model the proposed structures, which are created using the simulation tool by defining thicknesses of different layers and other physical characteristics. The simulations are carried out to obtain the J-V characteristics. At a temperature of 300 °C, numerical simulations of TOSC and IOSC were performed using the AM1.5 spectra. In this simulation model, the Neumann boundary condition with four electrical and six excitonic boundary conditions is utilized. The parameters obtained from the J–V characteristics are the current density-voltage characteristics (J-V), which include the short-circuit current density (*J*_sc_), open-circuit voltage (*V*_oc_), fill factor (*FF*), and efficiency (*η*).

**Figure 3:**
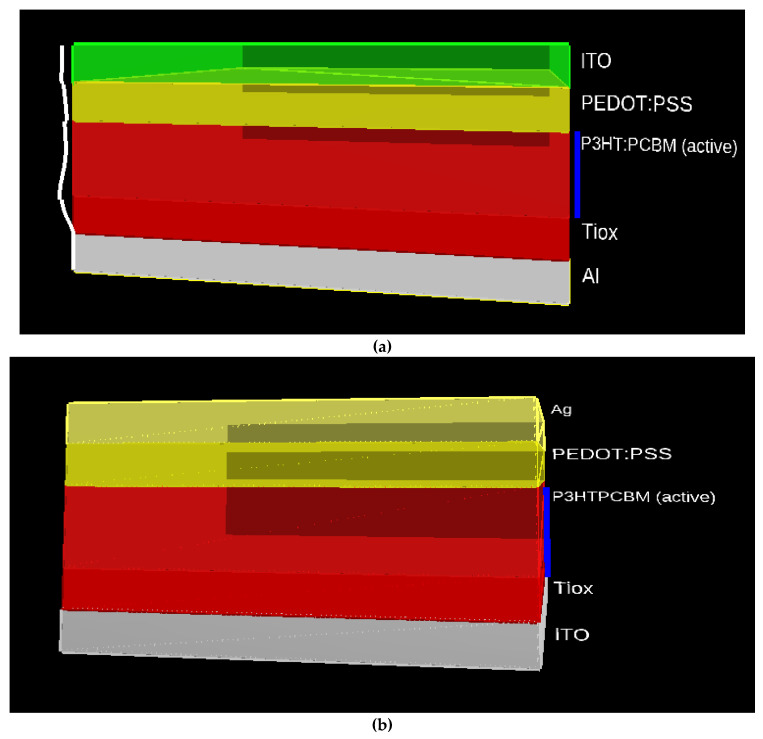
**(a**) TOSC and (**b**) IOSC BHJ structures used in this work.

Figure 3a,b depicts the NPD structure of TOSC and IOSC utilizing the BHJ structure, respectively. Figure 3a shows the organic solar cell structure (substrate/ITO/PEDOT:PSS/s-SWCNT:C_60_ (blended active layer)/TiOx/Al), which is a distinctive structure in which the cell is composed of an absorber layer (s-SWCNT:C_60_) and a hole transport layer (PEDOT: PSS). This assists the absorber layer in transporting charge carriers into an electron.

## 4. Results and Analysis

This work has demonstrated the hybrid architecture of TOSC and IOSC organic photodiode cells, which is composed of nanomaterials. This architecture allows them to optimize the different geometries of the devices and compare their outputs. To improve the performance of NPD devices, the TOSC and IOSC NPD cells with BHJ structures have been modeled. The results obtained have been compared to determine which structures have the best performance.

The properties of each solar cell have a role in increasing its PCE. The *V_oc_* is the difference in energy level between the potentials in the regions where free electrons and free holes predominate. The second parameter is *J_sc_*, the number of photogenerated carriers formed in the active layer upon illumination and the effectiveness of charge separation across the photoactive layer determines *J_sc_* [43,44]. As a result, to alter *J_sc_*, one needs to vary the structure that results in the lowest useable band-gap or alter the thickness of the active layer. The *FF* is the ratio of the maximum electrical power produced by the cell to the product *J_sc_ × V_oc_*. The *FF* influences the cell quality and corresponds to the *V_oc_*, *J_sc_*, and the shape of the active layer. The PCE is defined as the product of *V_oc_*, *J_sc_*, and *FF*. The relationship between cell electrical power and incident light power is represented by this measure [45,46].

The electrical device simulator has been used to mimic organic photodiode cells’ TOSC and IOSC architectures. The primary goal of these models is to optimize the anode layer thickness, active layer thickness, and cathode layer thickness to achieve the highest possible photocurrent density, the highest possible photovoltage, and efficiency. Consequently, the TOSC and IOSC device designs depicted in Figure 3 have been designed for simulation tests and the effects of various layer geometries on the above-mentioned device performance characteristics. The findings acquired for different structures have been compared to determine the optimal device dimensions for the device that provides the best overall performance.

### 4.1. Effect of Active Layer Thickness Variation

Optics, such as reflections at layer interfaces and accompanying interferences, cause complex enhancing difficulties that are proportional to the active layer thickness in the BHJ device. It is critical to analyze the active layer thickness since it affects light absorption and, consequently, the formation of excitons inside the active layer. In the BHJ device, layers are layered successively on top of one another, and optical events, such as reflections at layer interfaces and associated interferences, produce a complicated enhancing problem proportional to the active layer thickness [47]. The influence of active layer thickness on several parameters, such as *V_oc_*, *J_sc_*, *FF*, and *η*, has been evaluated and plotted against the thickness of the active layer for both TOSC and IOSC devices.

**Figure 4:**
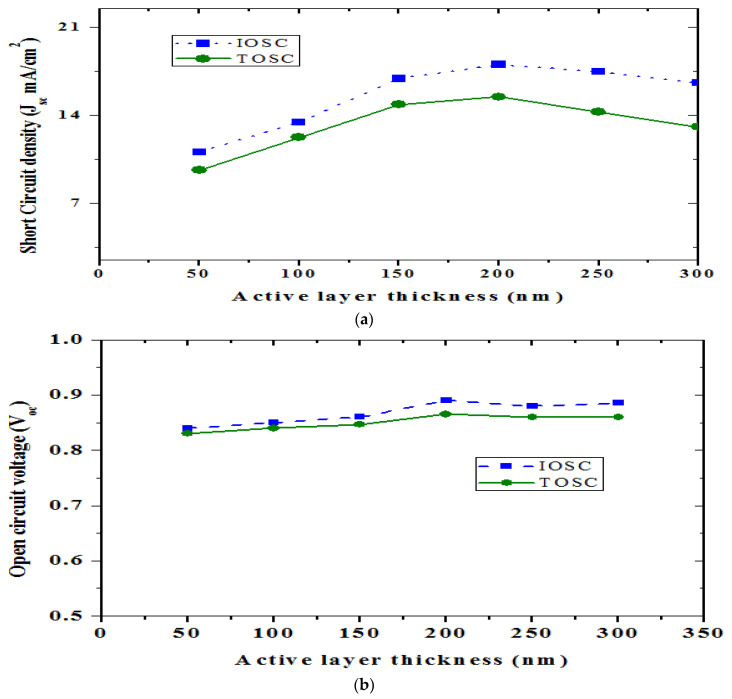

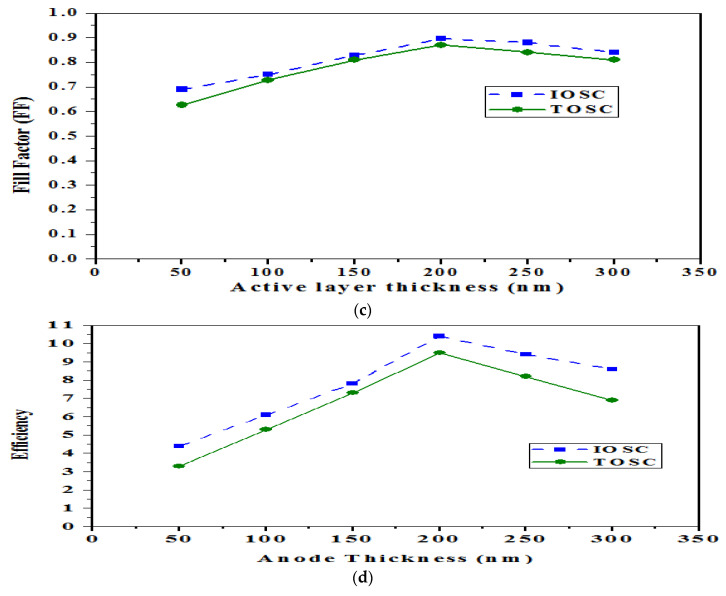
(**a**) *J_sc_*, (**b**) *V_oc_*, (**c**) FF, and (**d**) Efficiency. The output extracted (TOSC: Dashed lines and IOSC: Solid lines) for various active layer thicknesses is illustrated.

To conduct a comparative analysis and improve each BHJ structure’s performance, the active layer thickness varied from 50 to 300 nm. The author addresses organic photodiodes, in which the active layer thickness is changed between 50 and 300 nm. The anode layer 100 nm, HTL layer 50 nm, ETL layer 50 nm, and the cathode layer 100 nm remained constant. Figure 4 illustrates the comparison plot characteristics of TOSC (solid lines) and IOSC (dashed lines) for various active layer thicknesses from the JVV curves. The *J_sc_* characteristics are shown to be quite sensitive to changes in the active layer thickness. As previously stated, the *J_sc_* is significantly correlated with the active layer’s absorption. In both TOSC and IOSC, the thickness of the active layer has a considerable effect on *J_sc_*, *η*, and *FF*, while *V_oc_* appears to be less sensitive, as seen in Figure 4. Both the absorption and the diffusion length have an effect on the carrier photogeneration and transport as reflected by the *J_sc_* parameter.

In the TOSC, the best thickness for *J_sc_* is reached at around 200 nm, but in the IOSC, a saturation of the ideal *J_sc_* is found at approximately 200 nm. If there is a peak in the current curve at a certain thickness, it indicates thinner thicknesses and the absorption is not complete. Significant thicknesses make it more difficult for excitons to get through and weaken the internal field that keeps them apart, thus carrier collection will be less efficient. The active layer thickness is due to interferences between layers caused by optical phenomena, such as reflections at layer boundaries and their interactions; the active layer thickness is critical when optimizing BHJ devices. Since the thickness of the active layer affects the absorption of light and, consequently, the formation of excitons inside the active layer, the analysis of the active layer thickness is critical. The TOSC and IOSC have thinner active layers, with the active layer of TOSC as 200 nm and IOSC as 200 nm. The higher active layer thickness is due to the series resistance effect, whereas *V_oc_* drops marginally from 0.865 V in TOSC and IOSC to 0.89 V in both devices.

The *V_oc_* value is solely determined by the differential between the donor HOMO and acceptor LUMO, and there is a slight change in *V_oc_* by the active layer thickness. The *V_oc_* increases as the active layer thickness grows in TOSC and IOSC BHJ NPD, as indicated in Figure 4b. In *V_oc_*, there is no significant influence on active layer thickness fluctuation. When the applied bias voltage is zero, these values are produced. The connection between the number of excitons created and recombined during charge dissociation is represented by this value. The drop in *J_sc_* indicates that the active layer’s maximum number of excitons have recombined before reaching the p–n junction and that the recombination rate is complementary to *J_sc_*. As a result, when the active layer thickness increases in TOSC and IOSC, the current density drops (thickness above 200 nm for TOSC and 200 nm for IOSC). For both TOSC and IOSC, increasing the active layer thickness improves the current density (thickness from 50 to 200 nm for TOSC and 50 to 200 nm for IOSC). An increase in *J_sc_* indicates that the active layer’s maximal number of excitons reaches the electrodes and that the recombination rate is proportional to *J_sc_*.

**Figure 5:**
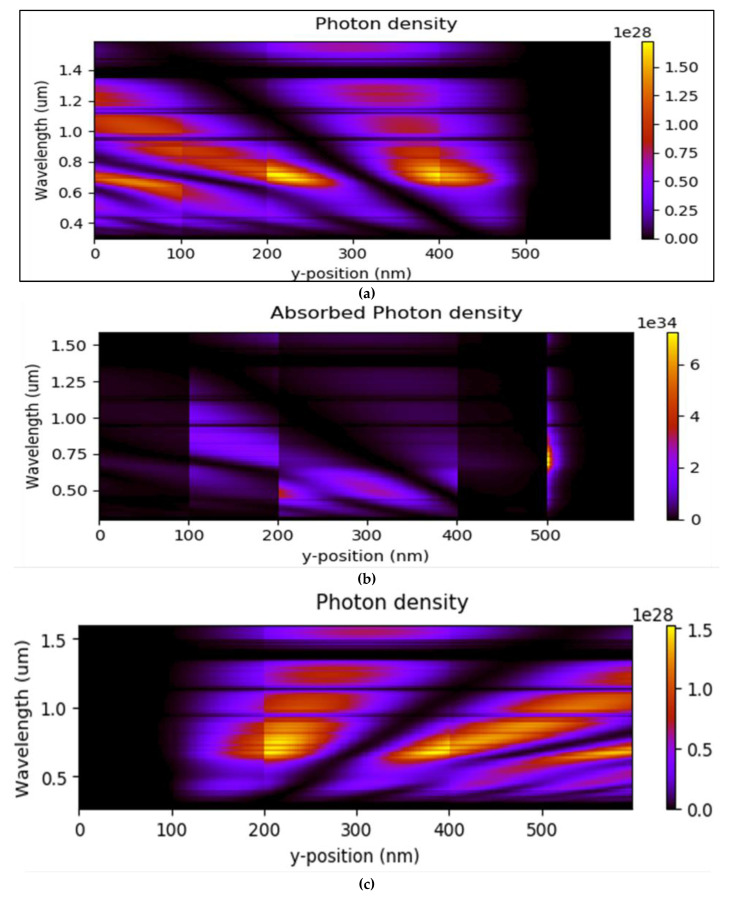

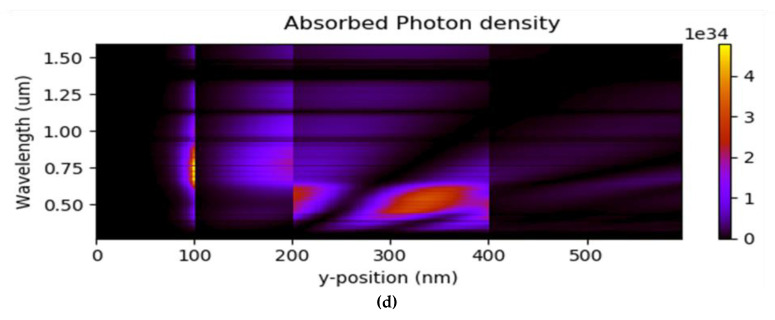
(**a**) Photon density of TOSC; (**b**) absorbed photon density TOSC; (**c**) photon density IOSC; (**d**) absorbed photon density IOSC. The output extracted using the transfer matrix method is illustrated.

As illustrated in Figure 4c, in both TOSC and IOSC NPD, the fill factor decreases slightly when the active layer thickness increases over 200 nm. This demonstrates that as the width of the I-V characteristic curves increases, the maximum power obtained decreases. The *FF* in both TOSC and IOSC BHJ NPD will grow as the active layer thickness increases from 50 to 200 nm; while above 200 nm it starts fall. Figure 5 shows the simulation output extracted from the transfer matrix model for both the TOSC and IOSC structures for the active layer thickness of 200 nm. Figure 5a,b shows the photon density and absorbed photon density of the TOSC structure with the active layer thickness of 200 nm. Additionally, Figure 5c,d shows the photon density and absorbed photon density of the IOSC structure with the active layer thickness of 200 nm.

When TOSC and IOSC are compared, the *J_sc_* of the IOSC is superior to the TOSC. This may be the case due to the light’s transmission and absorption properties. Initially, light passes through ITO, then through HTL PEDOT/PSS, and through contact on the HTL side to the active layer. However, the converse is true in the second case. Light reaches the active layer via the material’s *n*-component compact after passing through TiOx ETL [48,49,50]. Tunable White Light Transmission (TWFT) of the TiOx ETL is greater than the PEDOT/PSS HTL, which has lesser transparency to visible light than the PEDOT/PSS HTL 2.2 eV energy gap [51,52,53,54].

### 4.2. Optimized Performance of TOSC and IOSC Using s-SWCNT:C_60_ Active Layer

**Figure 6:**
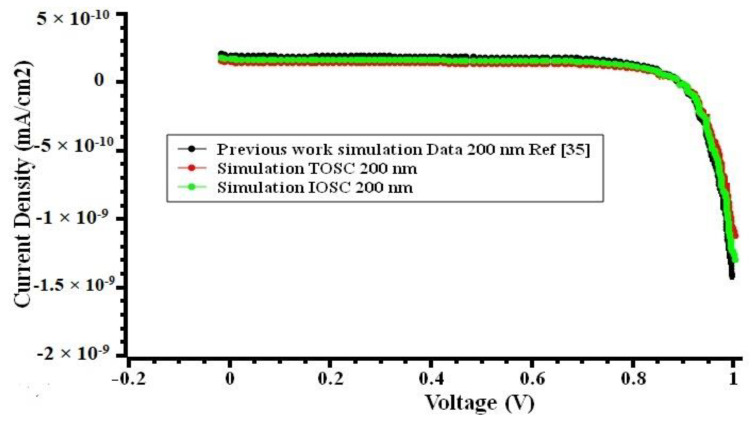
The J–V output plot of the IOSC (green line), TOSC (red line), and a previously reported work [35] (black line) of 200 nm thickness of active layer.

The results discussed above clearly demonstrated that IOSC BHJ NPD could provide superior performance than TOSC BHJ NPD devices for reasons elicited in the previous sections. The simulation results form the basis for the design of optimum geometries for the best performance. Of note, maximizing efficiency is the key to achieving the best possible performance. Table 1 shows the results of the foregoing analyses and comparison of results on different TOSC and IOSC NPD parameters, as well as the ideal active layer thicknesses for maximum performance. The best TOSC and IOSC performance parameters have been determined by running simulations with different parameters. The IOSC NPD device shows better results than TOSC NPD devices in all aspects. Figure 6 shows the J–V curve of the TOSC, IOSC, and a previously reported work [35] of 200 nm thickness of active layer.

## 5. Conclusions and Future Recommendations

Traditional and inverted s-SWCNT:C_60_ organic solar cells have been analyzed for enhanced performance. To design the TOSC and IOSC, a range of active layer thicknesses have been utilized. According to the findings, *V_oc_*, *J_sc_*, *η*, and *FF* were all evaluated and compared between TOSC and IOSC. In this research work, the active layer’s thickness was varied to observe how it affected the results. In both TOSC and IOSC, the thickness of the active layer has an effect on the overall performance. As a consequence, the maximum efficiency for TOSC is attained by employing the ITO/PEDOT:PSS/s-SWCNT:C_60_ (blended active layer)/TiOx/Al structure (efficiency of 9.5%). Another type of device (IOSC) has the most efficient structure, which was composed of ITO/TiOx/s-SWCNT:C_60_ (blended active layer)/PEDOT:PSS/Al. This device has a top-to-bottom structure and an efficiency of 10.4%. With respect to the active layers, the s-SWCNT:C_60_ displays the best power conversion efficiency, which was primarily attributed to its excellent optical and electrical characteristics, making it a strong contender in the role of the absorption layer.

This research proposes a viable way toward the effective deployment of OPV by adjusting their properties, which are heavily dependent on the results of organic solar cells to increase their efficiency. On the basis of the obtained data, it has been determined that the IOSC performance is superior to the TOSC in all categories. Finally, it has been concluded that IOSC NPD provides superior performance than TOSC.

In the future, this device will be optimized with fabrication using these nanomaterials. Thereafter, its characteristic will be verified with the simulated results. Moreover, the fabricated device has been implemented for future use in biomedical micro-device applications.

## Figures and Tables

**Table 1:** Optimum layer thicknesses for TOSC and IOSC NPD.

Parameter	TOSC	IOSC
Active layer thickness (nm)	200	200
Active layer material	s-SWCNT:C_60_	s-SWCNT:C_60_
*V_oc_* (V)	0.865	0.89
*J_sc_* (mA/cm^2^)	15.46	18.02
*η*	9.5	10.4
*FF*%	87	89.5

## Data Availability

All data and material used to prepare this manuscript are available in this document.
